# Patellar height changes after treatment of tibia plateau fractures: A radiological analysis

**DOI:** 10.15537/smj.2023.44.3.20220741

**Published:** 2023-03

**Authors:** Özgür Doğan, Ahmet Çulcu, İhsaniye Süer Doğan

**Affiliations:** *From the Department of Orthopaedics and Traumatology (Ö. Doğan), Ankara City Hospital, from the Department of Radiology (İ. Doğan), Ankara Dışkapı Yıldırım Beyazıt Education and Research Hospital, Ankara, and from the Department of Orthopaedics and Traumatology (Çulcu), Yüksekova State Hospital, Hakkari, Turkey.*

**Keywords:** plateau fractures, patellar height, Insall-Salvati index, Blackburne-Peel index, Caton-Deschamps index, Schatzker classification

## Abstract

**Objectives:**

To determine the impact of tibial plateau fractures on patellar height and the factors affecting this impact.

**Methods:**

A total of 40 patients treated for plateau fractures between 2017-2021 were evaluated in this retrospective prognostic study. The patient group consisted of lateral radiographs of the operated knees, whereas the control group consisted of lateral radiographs of the healthy sides of the same patients. Insall-Salvati, Caton-Deschamps, Blackburne-Peel, and modified Insall-Salvati indices were measured for both groups. In addition, Schaztker and Luo classifications, as well as the demographic profiles of the patients, were analyzed.

**Results:**

There was no significant difference between the groups in terms of patellar height indices (*p*>0.05). A significant relationship was found between the Insall-Salvati (*p*=0.046) and Blackburne-Pell (*p*=0.011) indices and Luo classification. Post hoc analyses revealed a significant relationship between the Insall-Salvati index and “One Column” fractures and between the Blackburne-Peel index and “Two Column” fractures.

**Conclusion:**

Long-term functions of tibial plateau fractures should be evaluated not only with a painless range of motion but also with patellar height. It should be noted that the Luo classification, which evaluates the plateau 3-dimensionally, may be associated with changes in postoperative patellar height values.

**S**urgical treatment is recommended for all tibial plateau fractures with a stepping of >3 mm, enlargement of >5 mm, varus/valgus instability of >10°, accompanying ligament injury, and bicondylar fractures.^1^ Surgical treatment options include open reduction and internal fixation, closed reduction and arthroscopic or fluoroscopy-guided internal fixation, and external fixation.

Regardless of the treatment method applied, achieving full knee function in the long term is one of the primary goals of treatment in patients with plateau fractures, along with anatomical reconstruction and stable fixation of the joint, and correction of the mechanical axis.^1-3^ Many scales and methods have been defined in the literature to evaluate the knee function of patients in the short, medium, and long terms.^2,4^ On the other hand, knee kinematics is not just regarding a painless range of motion; in particular, examination of the patellofemoral relation and patellar height is also very important in terms of knee function.^5^ Moreover, damage to the pes anserinus during the treatment of a plateau fracture, or fractures of the eminentia, or tibial tubercules may also cause patellar height-related disorders.

Patellar height plays an important role in the extensor mechanism, and contributes to knee stability.^5^ Patellar height disorders result in cartilage degeneration and instability, and the relationship between high tibial osteotomy, meniscal tears, tibia intramedullary nails, and patellofemoral instability has been exclusively investigated in the literature.^6-9^ However, studies on patellar height changes after treatment of plateau fractures are scarce.

We aimed to reveal the impact of tibial plateau fracture on patellar height and the factors affecting this impact by measuring patellar height using several indices, and comparing the index values with the healthy side of the patients. We hypothesized that patellar height indices would change and patients’ susceptibility to instability would increase after treatment of plateau fractures, especially in bicondylar fractures associated with high-energy injuries.

## Methods

In this retrospective prognostic study, following institutional review board approval of Ankara City Hospital No.1, Clinical Research Ethics Committee, Ankara, Turkey (issue number: E. Board-E1-22-2544, study number: 2544, date: 06.04.2022), patients who were treated for plateau fractures in the Orthopaedics and Traumatology Clinic in Ankara City Hospital, Ankara, Turkey, between October 2017 and September 2021 were examined. Informed consent was waived due to the retrospective nature of the study. To assess the impact of the treatment of plateau fractures on patellar height, patients who were treated with open reduction and internal fixation in our clinic and whose lateral radiographs of the intact knee were obtained at 30° semiflexion for any reason were included in the study. On the other hand, patients with known patellofemoral instability or a history of recurrent patella dislocation, patients who were operated on with closed reduction, staged treatment, or external fixation, and patients who did not comply with the recommended rehabilitation process, were excluded from the study. Following the inclusion and exclusion criteria, radiographs of 40 patients were analyzed and included in this study. The patient group consisted of lateral radiographs of the operated knees obtained in a 30° semi-flexed position at the last follow-up, while the control group consisted of lateral radiographs of the healthy side of the same patients obtained for any reason at any time in a 30° semi-flexed position. The study was carried out in accordance with the principles of the Helsinki Declaration.

In our clinic, the most preferred approach for the treatment of tibial plateau fractures with open reduction and internal fixation is the classical anterolateral approach, as described in the literature.^10,11^ In cases where the joint surface needs to be evaluated, submeniscal arthrotomy with reinforced sutures is carried out. A lateral buttress plate was applied with an anterolateral incision in all 40 patients included in our study. The classical posteromedial approach described in the literature is used in cases requiring posteromedial/posterior fixation.^10,11^ During this approach, the pes anserinus tendons are not completely loosened. A posteromedial/posterior plate was used in 17 (42.5%) patients. In terms of bicondylar fractures, although discussions regarding the optimal treatment continue in the literature, both single lateral plating and double plating are routinely carried out in our clinic.^1,2^ Although the preference usually depends on the presence of medial condyle coronal fracture/posteromedial column fracture, the condition of the soft tissue and the primary surgeon’s preference is also important.^2,12,13^

To evaluate the change in patellar height after treatment of plateau fractures, Insall-Salvati (IS), Caton-Deschamps (CD), Blackburne-Peel (BP), and modified Insall-Salvati (MIS) indices were measured on both the postoperative final follow-up radiographs and the 30° semi-flexed lateral radiograph of the intact knee obtained for any reason at any time and available in the computer archive system. All measurements were carried out by an experienced radiologist, using the hospital’s picture archiving communication system (SarusPACS, Teknoritma Software Services, Ankara, Turkey).

**Figure 1:**
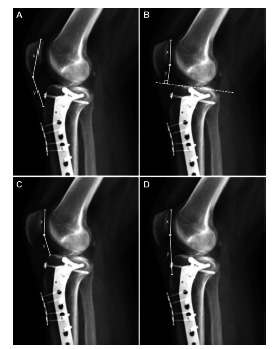
- Sample measurements of patellar height index values are seen on the X-ray of the patient who underwent lateral plate due to tibial plateau fracture. A second plate was applied for fixation of the anterior fragment of the patient’s tibia metaphysis. In terms of the measurements: A) Insall-Salvati index; B) Blackburne-Peel index; C) Caton-Deschamps index; D) Modified Insall-Salvati index

The IS index is the ratio of the patellar tendon length extending between the patella distal pole and the tuberositas tibia to the maximum length of the patella extending between the distal and proximal poles.^14,15^ The BP index is the ratio of the perpendicular distance of the distal pole of the patella to the plateau articular surface to the length of the patellar articular surface.^14,15^ The CD index is the ratio of the distance between the distal pole of the patella and the plateau anterosuperior to the length of the patellar joint surface.^14,15^ The MIS index is the ratio of the patellar tendon length extending between the distal pole of the patellar articular surface and the tuberositas tibia to the patellar articular surface length.^14,15^ The manner in which the measurements were carried out on an operated knee radiograph is shown in Figure 1. Although the normal values of the indices show social and racial differences, the generally accepted normal ranges are presented in Table 1.14,15

**Table 1:** - Normal range and clinically important values of patellar height indices.

Index name	Normal range	Patella baja	Patella alta
Insall-Salvati index	0.8-1.2	<0.8	>1.2
Blackburne-Peel index	0.5-1.0	<0.5	>1.0
Caton-Deschamps index	0.6-1.3	<0.6	>1.3
Modified Insall-Salvati index	1.2-2.1	<1.2	>2.1

In evaluating postoperative patellar height indices, because the patients did not have pre-injury radiographs in the system, index measurements and comparisons were carried out with the healthy side. Ideally, comparing the preoperative and postoperative values will reveal the changes more clearly. However, such a measurement is impossible in trauma patients, considering the sudden nature of trauma. Therefore, considering that the anatomies of the patients were identical between sides, comparisons were carried out with the opposite intact extremity, even though this view is controversial in the literature.^16^

Detailed analyses were carried out to reveal the impact of patient and fracture characteristics on the postoperative patellar height indices, by comparing the index values with gender, injury mechanism, Schatzker and Luo classifications, and presence of posteromedial/posterior plate. In these analyses, the mechanism of injury was examined under 3 headings (low-energy/simple fall, high-energy fall, and traffic accidents), and the Schaztker and Luo classifications were examined under 6 and 4 titles (Schatzker Type 1-6, Luo 0-3 Column Fracture), as indicated in the literature.^2,12,17^

### Statistical analysis

Statistical analysis was carried out using the Statistical Package for the Social Sciences, version 26.0 (IBM Corp., Armonk, NY, USA). The compliance of the data to normal distribution was determined using visual (histogram and probability plots) and analytical methods (Kolmogorov-Smirnov test). Considering the skewed distribution of the data, median, interquartile range, and minimum-maximum values were used as descriptive statistics, and the Wilcoxon signed-rank test was used to detect the difference between the averages. The Kruskal-Wallis test was used to compare data sets with 3 or more groups (injury mechanism, Schaztker, and Luo classifications). Post hoc analysis was carried out using the Mann-Whitney-U test between groups. A *p*-value of <0.05 was considered significant.

## Results

Among the 40 patients included in the study, 29 (72.5%) were male, and 11 (27.5%) were female, with a mean age of 49.3 years (range: 21-79 years). No patient had type 3 fractures according to the Schatzker classification, and “Zero Column” fractures were not detected in any patient according to the Luo classification. The average follow-up time of the patients was 15.1 months (range: 12-48 months). The detailed demographic profiles of the patients are shown in Table 2.

**Table 2:** - Demographic profile of the patients. N=40

Variables	n (%)
Age (years)	49.3 years (range: 21-79 years)
***Gender***	
Male	29 (72.5)
Female	11 (27.5)
***Side***	
Right	14 (35.0)
Left	26 (65.0)
***Injury mechanism***	
Simple fall	10 (25.0)
High-energy fall	8 (20.0)
Traffic accident	22 (55.0)
***Fracture type (Schatzker classification)***	
Type 1	12 (30.0)
Type 2	6 (15.0)
Type 4	6 (15.0)
Type 5	9 (22.5)
Type 6	7 (17.5)
***Fracture type (Luo classification)***	
One Column	21 (52.5)
Two Columns	12 (30.0)
Three Columns	7 (17.5)
***Posteromedial - posterior plate***	
None	23 (57.5)
Yes	17 (42.5)

Values are presented as numbers and precentages (%).

There was no significant difference between the postoperative patellar height indices: IS (*p*=0.248), BP (*p*=0.737), CD (*p*=0.845), and MIS (*p*=0.294) of the patients operated for tibia plateau fracture and the index values of the healthy knees (Table 3).

**Table 3:** - Patellar height indices of the patient and control groups.

Height indices	Patient group	Control group	*P*-values
Insall-Salvati index	0.738 (IR: 0.174)	0.745 (IR: 0.108)	0.248
	Range: 0.615-1.011	Range: 0.592-1.034	
Blackburne-Peel index	1.219 (IR: 0.279)	1.246 (IR: 0.21)	0.737
	Range: 0.882-1.685	Range: 0.806-2.047	
Caton-Deschamps index	1.065 (IR: 0.365)	1.027 (IR: 0.298)	0.845
	Range: 0.796-1.435	Range: 0.725-1.657	
Modified Insall-Salvati index	0.511 (IR: 0.078)	0.514 (IR: 0.64)	0.294
	Range: 0.413-0.644	Range: 0.458-0.598	

Values are presented as median (interquartile range [IR]) and minimum-maximum values were used as descriptive statistics for all skewed distributed variables.

Investigating the factors affecting postoperative patellar height index values, a significant relationship was found between the IS (*p*=0.046) and BP (*p*=0.011) indices and Luo classification. The relationship between postoperative indices and fracture characteristics is shown in Table 4. Looking at the detailed post hoc analyses, a significant relationship was detected between IS index and Luo “One Column” fractures and between the BP index and Luo “Two Column” fractures (Table 5).

**Table 4:** - The relationship of postoperative indices with fracture characteristics.

Variables	IS	BP	CD	MIS
***Gender***				
Female	0.739	1.221	0.996	0.532
Male	0.73	1.214	1.072	0.511
*P*-values	0.891	0.476	0.534	0.554
***Injury mechanism***				
Simple fall	0.739	1.256	0.897	0.537
High-energy fall	0.842	1.12	1.06	0.509
Traffic accident	0.73	1.267	1.136	0.522
*P*-values	0.181	0.98	0.574	0.605
***Schatzker classification***				
Type 1	0.697	1.323	1.049	0.498
Type 2	0.803	1.206	1.183	0.521
Type 4	0.725	1.307	1.169	0.514
Type 5	0.785	1.186	0.996	0.52
Type 6	0.763	1.235	1.036	0.542
*P*-values	0.68	0.639	0.056	0.538
***Luo classification***				
One Column	0.719	1.291	1.2	0.511
Two Columns	0.799	1.12	1.03	0.509
Three Columns	0.813	1.296	1.036	0.563
*P*-values	0.046	0.011	0.451	0.061
***Posteromedial - posterior plate***				
None	0.719	1.291	1.2	0.511
Yes	0.785	1.167	1.034	0.52
*P*-values	0.051	0.173	0.57	0.302

Median values were used as descriptive statistics for all skewed distributed variables. A *p*-value of <0.05 was defined as significant. IS: Insall-Salvati index, BP: Blackburne-Peel index, CD: Caton-Deschamps index,

MIS: Modified Insall-Salvati index

**Table 5:** - Post hoc analysis of the relationship between patellar height indices and Luo classification.

Luo classification	IS (*p*=0.046)*	BP (*p*=0.011)*
***Group one***		
One Column^†^	0.719	1.291
Two Column^†^	0.799	1.12
*P*-values^‡^	0.032	0.007
***Group 2***		
One Column^†^	0.719	1.291
Three Column^†^	0.813	1.296
*P*-values^‡^	0.066	0.576
***Group 3***		
Two Column^†^	0.799	1.12
Three Column^†^	0.813	1.296
*P*-values^‡^	0.933	0.018

* The relationship between Luo classification and IS index values were calculated using Kruskal-Wallis Test,

^†^ the median values of the groups were given,

^‡^ post hoc analyses between binary groups were analyzed using Mann Whitney-U test, IS: Insall-Salvati index,

BP: Blackburne-Peel index

## Discussion

Several studies have investigated the long-term functional outcomes of tibial plateau fractures.^1,2,18,19^ Milenkovic et al^19^ examined 41 lateral plateau fractures in 2021 and reported good-to-excellent results in 34 (82.9%) patients. Gencer et al^2^ investigated the results of bicondylar plateau fractures in 2022 using an accelerometer and reported satisfactory objective functional results and active behavioral patterns. However, all these studies were based on patients’ subjective functional results evaluated through predetermined questionnaires and scales and joint range of motion and muscle strength. Unfortunately, patellar height, which is a very important part of the functions and features of the knee joint, is not included in most of these scales. Studies examining the relationship between plateau fractures and patellar height disorders and associated patellar instability in the medium to long term are lacking. This constitutes the main strength of the present study. The most important finding of our study was that a significant relationship was found between Luo “One Column” fractures and Insall-Salvati index (*p*=0.046) and Luo “Two Column” fractures and Blackburne-Peel index (*p*=0.011).

According to the generally accepted values in the literature, patella baja was detected according to the IS index in 28 (70%) knees in the control group. Moreover, 27 (67.5%) knees in the patient group had patella baja according to the IS index, and 36 (90%) knees had patella alta according to the BP index. However, none of the patients had a history of patellar instability or associated clinical projection in either knee. We believe that the underlying reason for this is the difference in the interpretation of the cutoff values of the patellar height indices. The main reasons for these differences in interpretations are that different indices have different inter- and intra-observer reliabilities; all measurements were carried out on direct radiography in our study; and most importantly, although the cutoff values of patellar height indices have been defined in the literature, these values show racial and regional differences.^5,20,21^ For all these reasons, our study was based on the index values of the height indices of the patients rather than the interpretations. In addition, it should not be forgotten that these reference values are prepared for completely healthy individuals, and not for patients with treated lower extremity fractures.

Many studies have shown that fragmentation, increased displacement, and management of fracture configuration play important roles in fracture healing and are poor prognostic factors among different types of fractures.^22-24^ While establishing our hypothesis, we predicted that it would not be possible to achieve full knee function in plateau fractures with complex fracture patterns; therefore, there may be a problem with patellar balance in these patients. Similarly, Palke et al^25^ followed 73 patients treated 12 months and reported changes in gait patterns in all patients. However, no significant difference was found between the patellar height indices of the patient and control groups in our study (*p*>0.05 for each). This finding contradicts our hypothesis. As a result of this finding, it can be concluded that high-energy injuries and complex plateau fractures do not affect patellar height. However, the small number of patients in our study may have affected our results. Different results can be obtained with studies performed with a larger number of patients and in which subgroup analyses of fracture patterns associated with patellar balance (tibial tubercle fracture and tibia eminentia fracture) are carried out. Moreover, although no significant relationship was found between the injury mechanism and Schatzker classification and patellar height indices in our study (*p*>0.05 for each), a significant relationship was found between the Luo classification and IS indice (*p*=0.046) and BP indice (*p*=0.011). While Schatzker and other classification systems evaluated the tibial plateau as 2-dimensional, Luo et al^17^ evaluated the plateau as 3-dimensional. A 3-dimensional evaluation of the tibial plateau inevitably means that patellar balance and patellar height are also included in the evaluation. Thus, there is an expected significant relationship between the Luo classification and index values, which supports our hypothesis at some point. Post hoc analyses showed that the IS index was significantly lower (Table 5) in “One Column” fractures and the BP index was significantly lower in “Two Column” fractures. However, there is an obvious relationship between the Luo classification and patellar height, the extent to which this relationship has been established and its underlying causes remain unknown. Therefore, anatomical cadaver studies and biomechanical finite element analyses are needed to demonstrate this relationship and plan treatment according to its clinical projection.

### Study limitations

First, the relatively low number of patients and the retrospective design of our study are important limitations. With prospective study designs to be carried out with larger patient numbers, the effect of Luo classification on patellar height will be understood more clearly, and subgroup analyses can be carried out with tibial tubercle and eminentia fractures. Moreover, the clinical results and radiological findings of the patients can be compared; thus, the radiological results of patellar instability can be correlated with clinical findings. Second, as mentioned before, interpretations of the indices could not be evaluated due to racial and regional cutoff value differences. This limitation can be eliminated through multicenter and multinational studies. Another option is to determine regional and racial cutoff values for all patellar height indices and interpret the results accordingly. Another very important limitation was the combined evaluation of low- and high-energy plateau fractures in our study. Different results can be obtained with a subgroup analysis that evaluates these groups separately. On the other hand, we could not carry out subgroup analysis due to the relatively small number of patients in our study and the inhomogeneity of the patient numbers of the groups (10 patients after low-energy simple falls and 30 patients after high-energy injuries). On the other hand, assuming that low-energy injuries are associated with simpler fracture types while high-energy injuries are generally associated with complex fractures, we believe that looking at the relationship between fracture type and patellar balance could compensate for this limitation. Finally, although there are examples in the literature, it is an important limitation that all measurements were carried out only on direct radiographs and not on magnetic resonance or computed tomography images.

In conclusion, the long-term functions of tibial plateau fractures should not only be evaluated with a painless and adequate flexion-extension range of motion but also with patellar height and related patellar instability. In addition, it should be noted that the types of Luo classification, which evaluates the plateau 3-dimensionally, may be associated with changes in postoperative patellar height values.

## References

[1] Doğan Ö , Sapmaz U , Çalışkan E , Gencer B , Biçimoğlu A. Functional and radiological comparison of single or dual plate in Bicondylar tibia plateau fractures. Medeniyet Med J 2017; 32: 73–79.

[2] Gencer B , Doğan Ö , Çalışkan E , İğdir V , Biçimoğlu A. Single versus double plating for bicondylar tibia plateau fractures: comparison of range of motion, muscle strength, clinical outcomes, and accelerometer-measured physical activity levels. Knee 2022; 34: 187–194. 34959135 10.1016/j.knee.2021.12.002

[3] Mthethwa J , Chikate A. A review of the management of tibial plateau fractures. Musculoskelet Surg 2018; 102: 119–127. 29043562 10.1007/s12306-017-0514-8

[4] Fakıoğlu RA , Gencer B , Utkan A. Comparison of results of 3 different patient-based assessment scales in surgically treated adult ankle fractures. Çukurova Med J 2022; 47: 638–651.

[5] Igoumenou VG , Dimopoulos L , Mavrogenis AF. Patellar height assessment methods: an update. JBJS Rev 2019; 7: e4. 10.2106/JBJS.RVW.18.00038 30624307

[6] Carissimi M , Sautet P , Charre D , Hanak L , Ollivier M , Micicoi G. Patellar height is not modified after isolated open-wedge high tibial osteotomy without change in posterior tibial slope. Orthop Traumatol Surg Res 2021; 107: 103032. 34358712 10.1016/j.otsr.2021.103032

[7] Vampertzis T , Barmpagianni C , Nitis G , Papastergiou S. A study of the possible effect of abnormal patella height on meniscal tears. J Orthop 2020; 22: 170–172. 32419759 10.1016/j.jor.2020.04.012 PMC7215109

[8] Salem KH , Sheth MR. Variables affecting patellar height in patients undergoing primary total knee replacement. Int Orthop 2021; 45: 1477–1482. 33277664 10.1007/s00264-020-04890-6 PMC8178142

[9] Thompson P , Metcalfe AJ. Current concepts in the surgical management of patellar instability. Knee 2019; 26: 1171–1181. 31787447 10.1016/j.knee.2019.11.007

[10] Prat-Fabregat S , Camacho-Carrasco P. Treatment strategy for tibial plateau fractures: an update. EFORT Open Rev 2017; 1: 225–232. 28461952 10.1302/2058-5241.1.000031 PMC5367528

[11] Cho JW , Kim J , Cho WT , Kim JK , Samal P , Gujjar PH , et al. Approaches and fixation of the posterolateral fracture fragment in tibial plateau fractures: a review with an emphasis on rim plating via modified anterolateral approach. Int Orthop 2017; 41: 1887–1897. 28735430 10.1007/s00264-017-3563-6

[12] Kfuri M , Schatzker J. Revisiting the Schatzker classification of tibial plateau fractures. Injury 2018; 49: 2252–2263. 30526924 10.1016/j.injury.2018.11.010

[13] Lee AK , Cooper SA , Collinge C. Bicondylar tibial plateau fractures: a critical analysis review. JBJS Rev 2018; 6: e4. 10.2106/JBJS.RVW.17.00050 29461986

[14] Verhulst FV , van Sambeeck JDP , Olthuis GS , van der Ree J , Koëter S. Patellar height measurements: Insall-Salvati ratio is most reliable method. Knee Surg Sports Traumatol Arthrosc 2020; 28: 869–875. 31089790 10.1007/s00167-019-05531-1

[15] Yılmaz B , Ozdemir G , Sirin E , Cicek ED , Anıl BS , Bulbun G. Evaluation of patella alta using MRI measurements in adolescents. Indian J Radiol Imaging 2017; 27: 181–186. 28744079 10.4103/ijri.IJRI_222_16 PMC5510316

[16] Schenk P , Vlachopoulos L , Hingsammer A , Fucentese SF , Fürnstahl P. Is the contralateral tibia a reliable template for reconstruction: a 3-dimensional anatomy cadaveric study. Knee Surg Sports Traumatol Arthrosc 2018; 26: 2324–2331. 27872989 10.1007/s00167-016-4378-5

[17] Luo CF , Sun H , Zhang B , Zeng BF. Three-column fixation for complex tibial plateau fractures. J Orthop Trauma 2010; 24: 683–692. 20881634 10.1097/BOT.0b013e3181d436f3

[18] Verona M , Marongiu G , Cardoni G , Piras N , Frigau L , Capone A. Arthroscopically assisted reduction and internal fixation (ARIF) versus open reduction and internal fixation (ORIF) for lateral tibial plateau fractures: a comparative retrospective study. J Orthop Surg Res 2019; 14: 155. 31126304 10.1186/s13018-019-1186-x PMC6534860

[19] Milenkovic S , Mitkovic M , Mitkovic M , Stojiljkovic P , Stojanovic M. Lateral tibial plateau fractures-functional outcomes and complications after open reduction and internal fixation. Int Orthop 2021; 45: 1071–1076. 32740756 10.1007/s00264-020-04763-y

[20] Biedert RM , Tscholl PM. Patella Alta: a comprehensive review of current knowledge. Am J Orthop (Belle Mead NJ) 2017; 46: 290–300. 29309446

[21] Chandru AK , Ganesan GR. Measurement of Insall Salvati ratio and modified Insall Salvati ratio to assess the position of the patella in South Indian population. Int J Res Orthop 2017; 3: 23–25.

[22] Mthethwa J , Chikate A. A review of the management of tibial plateau fractures. Musculoskelet Surg 2018; 102: 119–127. 29043562 10.1007/s12306-017-0514-8

[23] Gencer B , Doğan Ö , Igdir V , Çulcu A , Caliskan E , Biçimoğlu A. Searching for a new parameter in the healing of tibia pilon fractures: fracture area measurement. J Am Podiatr Med Assoc 2022; 112: 20–137. 10.7547/21-137 33734386

[24] Jiang L , Zheng Q , Pan Z. What is the fracture displacement influence to fracture non-union in intramedullary nail treatment in subtrochanteric fracture? J Clin Orthop Trauma 2018; 9: 317–321. 30449978 10.1016/j.jcot.2018.04.002 PMC6224685

[25] Palke L , Schneider S , Karich B , Mende M , Josten C , Böhme J , et al. Anti-gravity treadmill rehabilitation improves gait and muscle atrophy in patients with surgically treated ankle and tibial plateau fractures after one year: a randomised clinical trial. Clin Rehabil 2022; 36: 87–98. 34355605 10.1177/02692155211037148

